# Color Fusion Effect on Deep Learning Classification of Uveal Melanoma

**DOI:** 10.21203/rs.3.rs-3399214/v1

**Published:** 2023-11-08

**Authors:** Xincheng Yao, Albert Dadzie, Sabrina Iddir, Mansour Abtahi, Behrouz Ebrahimi, David Le, Sanjay Ganesh, Taeyoon Son, Michael Heiferman

**Affiliations:** University of Illinois Chicago; University of Illinois Chicago

## Abstract

**Background:**

Reliable differentiation of uveal melanoma and choroidal nevi is crucial to guide appropriate treatment, preventing unnecessary procedures for benign lesions and ensuring timely treatment for potentially malignant cases. The purpose of this study is to validate deep learning classification of uveal melanoma and choroidal nevi, and to evaluate the effect of color fusion options on the classification performance.

**Methods:**

A total of 798 ultra-widefield retinal images of 438 patients were included in this retrospective study, comprising 157 patients diagnosed with UM and 281 patients diagnosed with choroidal nevus. Color fusion options, including early fusion, intermediate fusion and late fusion, were tested for deep learning image classification with a convolutional neural network (CNN). Specificity, sensitivity, F1-score, accuracy, and the area under the curve (AUC) of a receiver operating characteristic (ROC) were used to evaluate the classification performance. The saliency map visualization technique was used to understand the areas in the image that had the most influence on classification decisions of the CNN.

**Results:**

Color fusion options were observed to affect the deep learning performance significantly. For single-color learning, the red color image was observed to have superior performance compared to green and blue channels. For multi-color learning, the intermediate fusion is better than early and late fusion options.

**Conclusion:**

Deep learning is a promising approach for automated classification of uveal melanoma and choroidal nevi, and color fusion options can significantly affect the classification performance.

## Introduction

Uveal melanoma (UM) and choroidal nevi are melanocytic choroidal tumors that share similar clinical characteristics, presenting a formidable challenge in differentiating them^1,2^. It is however imperative to distinguish these two lesions for appropriate management. UM is a malignant tumor originating from the melanocytes of the uveal tract and is the most common primary intraocular malignancy in adults^3,4^. Approximately half of all UM patients develop distant metastasis during the course of their disease progression^5^. Choroidal nevi, although benign in nature, may exhibit features resembling UM and can rarely undergo malignant transformation^6^. Erroneous classification of these benign tumors as malignant lesions can lead to unwarranted treatments, such as radiation therapy or enucleation. Conversely, the misdiagnosis of UM as a benign lesion may have serious consequences, including greater vision impairment, a higher risk of metastasis and death^7^.

Biopsy, followed by histological investigation is considered the gold standard for tumor diagnosis^8,9^. However, in the context of ocular tumors, this gold standard approach is not applicable due to the potential risk of unnecessary ocular biopsy for benign tumors^10,11^. As a result, alternative diagnostic methods and imaging modalities are favored to avoid the risks associated with invasive procedures and to ensure the safety and integrity of the ocular structures. Prominent among these are fundus photography, fluorescein angiography (FA), optical coherence tomography (OCT), and ultrasonography (US). These imaging techniques are widely employed in the diagnosis and differentiation of UM and choroidal nevi, as they offer valuable insights into the anatomical and clinical characteristics of these ocular lesions. These methods, however, are not widely available at all eye clinics and often require an evaluation by an ocular oncologist to make a conclusive diagnosis. Moreover, the overlapping characteristics between these tumors exacerbate the diagnostic complexity, leading to inconclusive results and delays in appropriate management decisions.

Given these challenges, machine learning and deep learning offer promising solutions for automating the detection and classification of these tumors. These techniques have been successfully implemented in ophthalmology for the automated diagnosis of age-related macular degeneration (AMD)^12,13^, diabetic retinopathy (DR)^14–16^, sickle cell retinopathy^17,18^ and glaucoma^19–21^. However, there is limited utilization of machine learning and deep learning in the diagnosis of ocular tumors like UM and choroidal nevi^22^. Large amounts of data are often required for the development of accurate and reliable machine learning and deep learning models for automated disease diagnosis. However, due to the relative rarity of ocular tumors, gathering sufficient data for this purpose becomes challenging. This scarcity of available data could be a contributing factor to the limited research on this subject matter. Nonetheless, the promise shown by machine learning and deep learning techniques in other ophthalmic applications sparks considerable potential for diagnosis and classification in ocular oncology.

One approach that holds promise in overcoming the challenge of limited data is transfer learning. Transfer learning is a training method that leverages the pre-trained weights of a convolutional neural network (CNN) on a large and diverse dataset and fine-tunes specific layers to adapt them for a new and more specific task. By utilizing the knowledge learned from the initial training on the broader dataset, transfer learning aims to optimize the weights of the CNN for the targeted task, enabling the model to excel in specialized applications, even with limited amounts of data available. This technique has proven to be highly effective in various applications in ophthalmology^23,24^. Some prior studies have also explored the combination of transfer learning with data fusion in ophthalmology^16,25–27^.

Data fusion is a powerful strategy widely employed to enhance the performance of deep learning models. This technique involves integrating data from multiple sources or modalities and has applications in several fields. Notably, in ophthalmology, data fusion has been employed both using the same imaging modality ^16,27,28^ or different imaging modalities^25,26^ for the automated diagnosis of retinal diseases. By fusing diverse data streams, the deep learning model gains access to a broader spectrum of information, allowing it to capture intricate patterns and correlations that may be challenging to discern using individual sources. There are three main strategies employed in the fusion of data for deep learning applications: early, intermediate, and late. In the early fusion strategy, the data is combined before any processing is done while in the intermediate fusion strategy, representations of the individual data are merged at an intermediate stage of the network. Late fusion, also known as decision-level fusion, combines predictions from individual modalities at the final layer of the network.

In this study, we explored the feasibility of employing a transfer learning approach along with data fusion strategies for automated classification of UM and choroidal nevi. To enhance the accuracy of classification, we incorporated early, intermediate, and late fusion strategies on distinct color channel images. This approach aimed to harness the unique information offered by each color channel – with red potentially providing crucial tumor-related information, while blue and green channels potentially contributing insights into features such as drusen, orange pigment and subretinal fluid. Our objective was to determine the most effective color fusion strategy for accurately differentiating UM and choroidal nevi.

## Methods

### Study Population

In this retrospective study, we investigated the feasibility of using deep learning and color fusion strategies for automated classification of UM and choroidal nevi using ultra-widefield retinal images (Optos PLC, Dunfermline, Fife, Scotland, UK). The study was approved by the Institutional Review Board (IRB) of the University of Illinois at Chicago (UIC) and was conducted in accordance with the guidelines in the Declaration of Helsinki. The study cohort consisted of 438 patients who were diagnosed with melanocytic choroidal tumors, comprising 157 cases of UM and 281 cases of choroidal nevus. These patients were seen and clinically diagnosed at the University of Illinois at Chicago (UIC) eye clinic between January 2010 and July 2023. Patients who had been treated prior to presentation were excluded from the study. For each patient included in the study, ultra-widefield retinal images were obtained from both eyes: the eye with the tumor and the fellow eye, which served as the control. Fellow eyes with opaque ocular media or a choroidal tumor were excluded from this study.

For the training and validation of the model, a total of 798 ultra-widefield retinal images were used. Among these, 157 images were from patients diagnosed with UM, and 281 images were from patients diagnosed with choroidal nevus. Additionally, the dataset included 360 images from the fellow eye of 360 patients with either UM or choroidal nevus who met the study’s eligibility criteria. All images were directly fed into the deep learning model without undergoing any preprocessing techniques such as histogram equalization, contrast adjustments, or cropping. This deliberate decision was made to ensure seamless integration of the model used in this study into future clinical settings.

### Deep Learning Architecture

The DenseNet121 was selected as the base CNN architecture for this study due to its success in the field of ophthalmology for the classification of several ocular disorders^25,29–31^. The base architecture is used for feature extraction from the input images. The extracted features are then passed through 3 fully connected dense layers for classification into either control, UM or choroidal nevus. The transfer learning approach was employed to mitigate the problem of limited data. Pretrained weights from the ImageNet dataset were used to initialize the model for transfer learning.

All deep learning implementations were carried out using the Keras API with Tensorflow backend. To mitigate the risk of overfitting and enhance the generalizability of the model, data augmentation techniques, including horizontal and vertical flips, as well as rotations were applied. These augmentations served to diversify the training data, enabling the model to learn diverse variations of the training data and improving its ability to make accurate predictions on unseen images. The training of the model was done on a Windows 11 computer (Microsoft, Redmond, WA, USA) using the NVIDIA RTX A6000 graphics processing unit (Santa Clara, CA, USA). The training procedure employed the Adam optimizer with a learning rate of 0.00001 and a batch size of 32. To ensure robust evaluation and reliable performance assessment, a five-fold cross-validation procedure was conducted. By averaging the results across all five folds, we obtained a comprehensive and statistically sound evaluation of the model’s classification accuracy, providing robust insights into its overall performance. For each fold, 80% of the images were used for training while the remaining 20% of the images were used for validation of the model’s performance.

**Figure 1:**
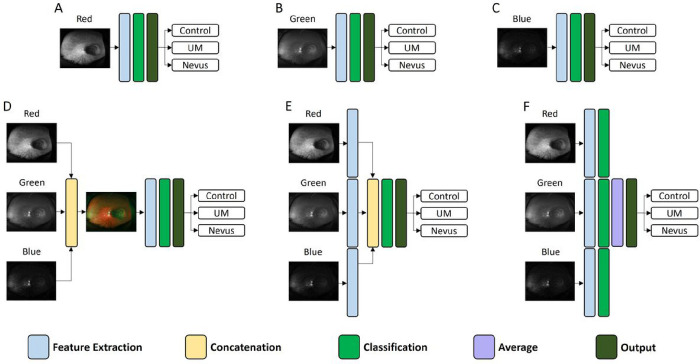
Fusion Strategies A. Red channel only. B. Green channel only C. Blue channel only. D. Early fusion strategy E. Intermediate fusion strategy. F. Late fusion strategy.

Initially, our approach involved evaluating the performance of the model using single-channel (red, green, or blue) images as the input. Subsequently, we assessed the effectiveness of different color fusion strategies in enhancing classification accuracy. The three fusion strategies used in this study consisted of early fusion, intermediate fusion, and late fusion. A visual representation of the fusion strategies is shown in Fig. 1.

### Early Fusion

Early fusion, also known as input-level fusion, is a fusion strategy that merges different data at the input level of the model. In our implementation, early fusion entailed using the exported images directly from the Optos device, where red, green, and blue channels served as separate and distinct input channels. Figure 1D shows how the early fusion strategy was implemented in this study. By combining the information from the different color channels, the model is able to capture intricate color patterns and correlation that may not be discernible when processing each color channel image independently.

### Intermediate Fusion

Intermediate fusion is another fusion strategy employed in deep learning where data fusion occurs after some initial processing. With this strategy, each color channel serves as an input to a separate classification branch. The feature extractors process the individual channels independently, extracting relevant features from each branch. Subsequently, the extracted features are merged before being fed into the classification layers for decision making. A representation of this process is illustrated in Fig. 1E.

In this study, we employed the intermediate fusion strategy by feeding the individual color channels into distinct feature extractors. Each channel underwent separate processing to extract distinctive and relevant features. These extracted features were then concatenated and passed on to the fully connected dense layers, allowing the model to exploit the combined information from the different channels to make more informed and accurate classification decisions. This approach allows the model to capture complementary features from each channel and consequently enhances the model’s classification performance.

### Late Fusion

Late fusion or decision-level fusion is a strategy that fuses the decision results from the independent channels while ignoring any correlation between the different channels. In this approach, the predictions from each separate classification branch are combined at the final layer of the model. A visual presentation of this process is illustrated in Fig. 1F. This approach enables the model to leverage the individual strengths of each color channel to make the final classification decision. While this approach may not directly consider any potential interdependencies between the channels, it allows the model to access a comprehensive range of predictions for each image, potentially leading to improved classification performance.

### Performance Evaluation

To ensure robust evaluation, we conducted a five-fold cross-validation procedure. The model’s performance was assessed by averaging the results across all five folds, providing a comprehensive and reliable evaluation of its classification accuracy. Five evaluation metrics were used to assess the models’ performance: specificity, sensitivity, F1 score, accuracy, and the area under the curve (AUC) of a receiver operating characteristic (ROC).

Specificity measures the proportion of true negative predictions among all the actual negative cases, while sensitivity, also known as recall, represents the proportion of true positive predictions among all the actual positive cases. The F1 score combines both precision and recall, providing a balanced assessment of the model’s performance. Accuracy reflects the overall correctness of the model’s predictions across all classes and is calculated as the proportion of correct predictions over the total number of instances. The AUC represents the area under the ROC curve, providing a measure of the model’s ability to discriminate between different classes effectively.

### Saliency Map Visualizations

The gradient-weighted class activation mapping (Grad-CAM)^32^ technique, a widely used visualization tool in deep learning, was employed in this study to gain insights into the information utilized by the model for making classification decisions. Grad-CAM offers an interpretable visualization of the regions within the input images that significantly influence the model’s final prediction. This visualization tool provides valuable interpretability and contributes to transparency and trust in the automated classification process.

## Results

A total of 157 ultra-widefield retinal images from patients diagnosed with UM and 281 images from patients diagnosed with choroidal nevi were used in the study. A total of 360 images from the fellow eyes of these patients that met the eligibility criteria for this study were also included to serve as controls. A summary of the characteristics of the patients is shown in Table 1. The mean age in both groups was not significantly different. The study subjects were predominantly white, which is consistent with the observed prevalence of UM and choroidal nevi^3,33–36^.

**Figure 2:**
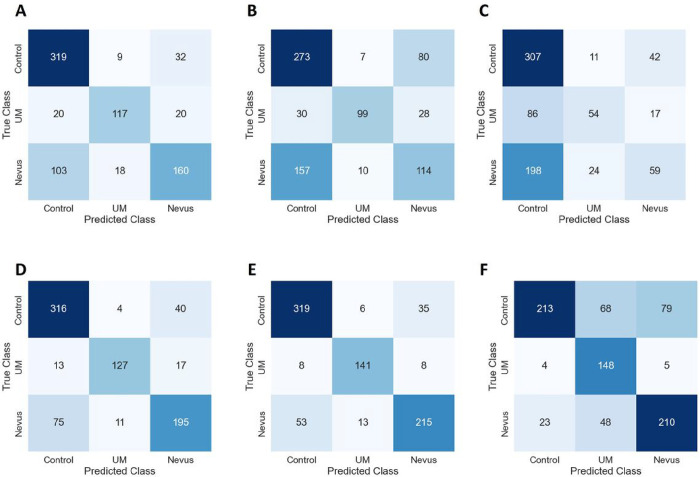
Confusion Matrices of the Models The confusion matrices of the various models: A. Red only, B. Green only, C. Blue only, D. Early fusion strategy, E. Intermediate fusion strategy, F. Late fusion strategy.

**Figure 3:**
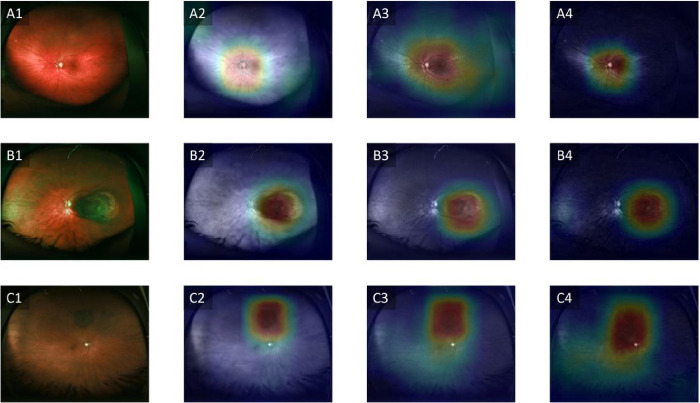
Class Activation Maps of the Intermediate Fusion Strategy Heatmap visualizations of the areas that were most important for the intermediate fusion strategy in classifying the various classes: A. Control, B. Uveal melanoma, C. Choroidal nevus. Column 1 represents the original images, column 2 represents the red channels, column 3 represents the green channels and column 4 represents the blue channels.

Table 2 shows the cross-validation evaluation metrics of the model used in this study. Using single color channel images, the red-only achieved the best performance across all metrics with an average specificity of 85.89%, sensitivity of 73.30%, F1 score of 0.7387, accuracy of 83.79% and an AUC of 0.8484. Using the fusion strategies, the intermediate fusion also achieved the best results across all metrics with an average specificity of 91.64%, sensitivity of 85.05%, F1-score of 0.8492, accuracy of 89.72% and an AUC of 0.9335. Figure 2 shows the confusion matrices of the various color channels and the color fusion strategies. For each approach, the confusion matrices from the five folds were combined to provide a comprehensive overview of the model’s performance. Figure 3 shows representative images of the various classes, and the class activation maps from the intermediate fusion model which emerged as the best-performing model. Remarkably, the model focused on the optic disc for normal images and the tumor location for UM or choroidal nevus.

## Discussion

Distinguishing UM and choroidal nevi present a notable diagnostic challenge due to their potentially subtle and overlapping clinical characteristics^1,2^. These similarities can lead to difficulties in accurate differentiation, referral decisions, and potentially impacting management decisions. To address this clinical dilemma and enhance diagnostic accuracy, we investigated the feasibility of employing deep learning techniques in combination with color fusion strategies for the objective and automated classification of these ocular lesions. Addressing the inherent challenge of limited image data, we leveraged the power of transfer learning.

Interpreting deep learning systems can be a challenge due to their inherent complexity. To address this issue, we employed a saliency map visualization technique known as Grad-CAM^32^. This method allowed us to pinpoint the specific regions within the input images that had the most significant impact on influencing the classification decisions made by the CNNs. The saliency maps obtained from this analysis reveal a consistent pattern across the various color channels and color fusion strategies, indicating a predominant focus on the tumor and its immediate surroundings for the classification process. This suggests that the models are able to identify key features associated with the presence of UM and choroidal nevi, highlighting the clinical relevance of these regions in diagnosing these ocular conditions.

For this study, we first evaluated the performance of the deep learning model to differentiate normal, UM and choroidal nevi eyes using single-channel images from the ultra-widefield retinal images. The results showed that the red channel achieved the best performance compared to the green and blue channels in all the performance metrics. This is because the red channel (635 nm) reveals the deeper structures from the retinal pigment epithelium (RPE) to the choroid, where both UM and choroidal nevi are located^37^. The blue channel (488 nm) allows visualization of anterior retinal layers, and the green channel (532 nm) captures information from the deeper layers of the retina to the RPE^37–39^. It is however interesting to note that although the green and blue channels do not capture information from the choroid, they achieved acceptable performance. This suggests that they carry complementary information from the inner and outer retina, which could potentially improve the classification performance if combined with the features from the red channel. To test this, we implemented and evaluated three different fusion strategies: early, intermediate and late.

Data fusion, which is widely employed to enhance the performance of deep learning models, involves the integration of data from multiple sources. The source of the data can be from the same modalities or different modalities. In this study, the data used were from the same modality, specifically color retinal images. While the fusion of multiple data modalities undoubtedly enriches diagnostic insights, the practicality of utilizing data from a single modality remains a compelling proposition. The streamlined process of using data from a singular source expedites diagnosis and simplifies data collection. It is also relatively cheaper to use a single modality, making it an attractive option for healthcare systems with limited resources. Therefore, by using a single modality for fusion strategies, not only do we achieve better diagnostic performance, but we also ensure a seamless transition into clinical practice. The results show that the fusion strategies employed in this study recorded better performance compared to the single-channel models. This observed improvement in performance can be attributed to the combination of complementary information from the different color channels. The red channel scans the deeper structures including the choroid where both UM and choroidal nevi are located. The blue and green channels contribute supplementary information, potentially capturing features like drusen, orange pigment, and subretinal fluid from different retinal layers. This combined information allows the models to make more informed and accurate distinctions between normal eyes, UM and choroidal nevi.

Among the fusion strategies, intermediate fusion achieved the best performance across all measured metrics. Characterized by the separate extraction of features from individual color channels followed by merging of these features before the classification layers, the intermediate fusion strategy allows each channel to contribute its own unique features. This approach capitalizes on the inherent strengths of each channel in capturing specific features relevant for classification decisions. In contrast, by combining the color channels at the input level, the early fusion only learns low-level features but may not capture complex inter-channel relationships, explaining its subpar performance compared to intermediate fusion^40^. Late fusion on the other hand, combines the decisions of the individual color channels to make a final decision. The decisions of individual color channels show a lack of direct correlation, and this might lead to possible complementary effects, which could explain why the late fusion outperformed the single channel approaches. However, late fusion might not be capable of learning the separate channel effects at the input or feature level, explaining its subpar performance compared to early and intermediate fusion.

The limitations of this study include its retrospective nature and relatively modest dataset size of images owing to the rarity of malignant ocular tumors. Also, the study was conducted in a single center which may limit the generalizability. Future studies from multiple centers using larger and more diverse datasets will help further validate the use of color fusion strategies for objective classification of melanocytic choroidal tumors.

## Conclusion

This study confirmed the feasibility of using deep learning for automated classification of UM and choroidal nevi. Color fusion options were observed to affect the deep learning performance significantly. For single-color learning, the red color image was observed to have superior performance compared to green and blue channels. For multi-color learning, the intermediate fusion is better than early and late fusion options. Automated classification of choroidal tumors holds a great promise to facilitate rapid diagnosis, triage referrals and prompt effective treatment.

## Figures and Tables

**Table 1:** Demographic characteristics of study participants

Characteristic	Uveal Melanoma (N = 157)	Choroidal Nevus (N = 281)
Age, mean (SD), y	67.5 (13.9)	63.2 (16.7)
Sex, No. (%)		
Male	72 (46)	99 (35)
Female	85 (54)	182 (65)
Race, No. (%)		
White	124 (84)	166 (64)
Black or African American	3 (2)	18 (7)
Asian	1 (1)	9 (4)
Other	19 (13)	65 (25)

**Table 2:** Cross-Validation Evaluation Metrics

Model	Specificity Mean % (SD)	Sensitivity Mean % (SD)	F1 Score Mean (SD)	Accuracy Mean % (SD)	AUC Mean (SD)
Red Only	85.89 (2.89)	73.30 (5.99)	0.7387 (0.0475)	83.79 (2.24)	0.8484 (0.0352)
Green Only	77.97 (4.22)	59.90 (7.05)	0.6119 (0.0516)	73.93 (3.04)	0.7258 (0.0418)
Blue Only	72.83 (8.80)	47.02 (13.21)	0.4407 (0.1208)	68.41 (4.75)	0.6436 (0.0775)
Early Fusion	88.76 (3.34)	79.17 (6.26)	0.8001 (0.0307)	86.63 (1.45)	0.8798 (0.0324)
Intermediate Fusion	**91.64 (3.57)**	**85.05 (5.58)**	**0.8492 (0.0472)**	**89.72 (3.25)**	**0.9335 (0.0266)**
Late Fusion	86.53 (3.25)	76.70 (4.77)	0.7130 (0.0535)	81.04 (3.57)	0.9160 (0.0529)
